# Liaison Old Age Psychiatry Service in a Medical Setting: Description of the Newcastle Clinical Service

**DOI:** 10.1155/2011/587457

**Published:** 2011-07-11

**Authors:** E. B. Mukaetova-Ladinska, G. Cosker, M. Coppock, M. Henderson, Y. Ali Ashgar, A. Hill, A. Scully, D. Robinson, K. Sells, S. Brotherton, C. Lowthian

**Affiliations:** ^1^Liaison Old Age Psychiatry Service, Campus for Ageing and Vitality, Newcastle upon Tyne NE4 6BE, UK; ^2^Institute for Ageing and Health, Campus for Ageing and Vitality, Newcastle upon Tyne NE4 5PL, UK; ^3^Old Age Psychiatry Services, Campus for Ageing and Vitality, Newcastle upon Tyne NE4 6BE, UK

## Abstract

Liaison Old Age Psychiatry services (LOAP) have begun to emerge in the UK and further development of the service is supported by the latest health policies. Since qualitative and quantitative studies in this area are lacking, we have undertaken a detailed quantitative prospective review of referrals to the Newcastle LOAP to evaluate the clinical activity of the service. We report high referral rates and turnover for the LOAP service. Reasons for referral are diverse, ranging from requests for level of care and capacity assessments and transfer to other clinical services to management of behaviour, diagnosis, and treatment. We outline the value of a multidisciplinary model of LOAP activity, including the important role of the liaison nursing team, in providing a rapid response, screening, and followup of high number of clinical referrals to the service.

## 1. Introduction

In contrast to liaison psychiatric services for adults, liaison services for older adults are still in their infancy. A recent review in the UK outlined that despite an increasing number of specialist teams, most of the services (73%) are provided by a generic, sector-based psychiatry model [1]. One of recent College documents Who Cares Wins (2005) [2] provides a comprehensive outlook on the mental health care for older people in general hospital settings, including liaison mental health teams. This document is in line with the National Service Framework for Older People [3] that calls for a skill-mix able to meet complex needs of older people. The liaison old age psychiatry (LPOA) services meet at least several of the NSF standards. Similar policy has now been accepted by the recent NICE guidelines for depression [4] that incorporate screening for depression in general medical hospitals. 

The role of psychiatric input in the medical care of elderly individuals in general medical settings has been further stressed in the document Everybody's Business (http://www.everybodysbusiness.org.uk/, 2005). This is a service development guide that sets out the key components of a modern older people's mental health service, aiming towards improving people's quality of life, meeting complex needs in a coordinated way, providing person-centred approach and promoting age equality. Similarly, the most recent National Dementia Strategy [5] also aims of achieving better awareness of dementia, early diagnosis and high quality treatment at whatever stage of the illness and in whatever setting. All these policy documents support the need for further development and implication of LOAP in providing mental health care in medical setting for the elderly population, in the light of the high admission rates of elderly to medical wards who also have high comorbidity with mental health problems [6]. 

Since very little work has been done on the composition of liaison services for older adults, their role, and professional input in clinical settings, we have undertaken a 5-year prospective study to address the clinical activity of the Newcastle Liaison Service for Older Adults.

## 2. Material and Methods

### 2.1. Description of Services

A newly integrated Liaison Old Age Psychiatry (LOAP) service was established in the Newcastle area (estimated population 41,000 over 65 years of age) in mid July 2005 following a service review. The service covers four hospital sites in the city and takes referrals from all wards providing medical/surgical care to elderly medically ill patients (currently 63 wards) and 2 Care of the Elderly Day Hospitals. 

The team consists of three full-time registered mental health nurses (RMNs; AH/MH, GC, and MC), one of whom is also a registered general nurse (GC), 0.8 administrator (YAA) and an equivalent of one consultant old age psychiatrist (EBM-L and AS, who are also specialists in neuropsychiatry and general medicine, resp.). We have *ad-hoc* consultations from the psychology service for older adults, but no input from occupational therapy or social work for our service.

### 2.2. Data Collection

**Figure 1:**
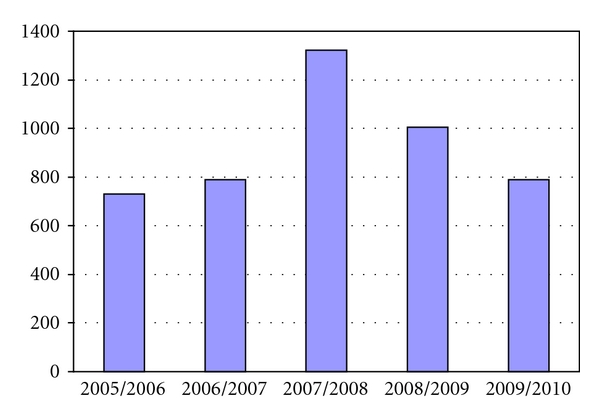
Annual referral rate over 5 years period to LOAP service. The decrease in referrals in 2008/2009 and 2009/2010 period reflects the restructuring of medical wards on the 4 hospital sites in Newcastle, as described in the text, and also the introduction of the Mental Capacity Act (discussed in greater detail in the text).

Data regarding LOAP activity has been continuously collected over the last 5 years, with an aim to monitor the team activity (Figure 1). During the last 2 years (since 2007/2008 to now), the Acute Medical and Primary Care Trusts have undergone organisational changes, with various medical wards being reorganised, and reduction of bed capacity. In particular, medical wards on two hospital sites, Newcastle General Hospital (now Campus for Ageing and Vitality) and Walkergate Hospital, were closed, and some of them were either transferred or amalgamated within existing medical wards on the other 2 hospital sites (The Royal Victoria Infirmary and the Freeman Hospital). In this paper, we report on the prospectively collected and analysed referrals over a period of 5 years, providing more detailed analysis regarding the nature of referrals and outcomes for 1 year period only (18 July 2005 to 17 July 2006). Please note data for 2007/2008 are not included in analysis, as a result of not fully completed audit for this period, due to LOAP service redevelopment in this period.

The information about the number of admissions on all 4-hospital sites during the same period was obtained from the Information Services Department, The Newcastle upon Tyne Hospitals NHS Foundation Trust. Parameters collected for analysis include: gender, age, reason for referral, source of referral, urgency of referral, number of follow-ups, diagnosis, response time, previous contact with psychiatric services, and current involvement with psychiatric services. Clinical diagnoses were based on DSM-IV criteria (1994) [7].

### 2.3. Statistical Analysis

For statistical analysis, nonparametric analysis was used (Chi-square test) to test the differences between sample proportions. We have also used percentage analysis to present data (a part in 100%) for better understanding of the collected data.

## 3. Results

### 3.1. Description of Clinical Service Activities

During five-year period, a total of 4637 referrals were made to LOAP (average of 927 referrals annually; 60% females and 40% males; Figure 1) which represent an average of 4.3% of all nonelective admissions in the Newcastle hospitals. The average age of patients referred to the service is 81 years old, the oldest being 104 and the youngest 60 years. Only a smaller portion of the referred patients were below the age of 65 years (average of *n* = 19 annually; 2.05%). The majority of these patients were referred from Stroke or Rehabilitation wards where there is currently no services provided by Adult services. 

The referrals were predominantly generated from acute medical (25.86%), care of the elderly (25.3%), rehabilitation (15.9%) wards, with only 2.3–5.5% (17/731–55/1005 for 2005/2006 and 2009/2010 year, resp.) from the two Geriatric Day Hospitals. These figures remain largely unchanged over the 5 year period service activity, with only marginal increase (19.9%; 157/789) in referrals from rehabilitation wards in 2010 year. Referrals to LOAP came from various sources: nursing staff (42.5%), medical staff (48.5%, mainly junior medical doctors, whereas the referral rate from consultants was 3.5%). 6% of the referrals to LOAP came from Old Age Psychiatry services in the Newcastle area, with only 3% from social services and medical occupational therapists.

### 3.2. Analysis of Clinical Assessments

#### 3.2.1. Response Time

 In the first year of the LOAP service activity, out of the 731 referrals to the service (2005-2006 period), the majority (85.3%) were seen within the first 2 working days (Table 1): 287 (39.3%) within the same day of referral, 209 (28.6%) the following day and 127 (17.4%) the second day. The remaining 108 patients were seen within the 3–5 working days (7.8% on the 3rd day, 4.2% on the 4th day, and 0.9% on the 5th day). Thus, 98.2% of the referrals were seen within 2–5 working days, and this response time has remained unchanged over the 5 years (2005–2010) (Table 1). However, over the last 2 years, 87%–93% of all referred patients were seen within one day in comparison to 68% for the first year (Table 1). On average 2% of patients tend to be seen after 5 days waiting period, and this is largely due to them being either too physically unwell or attending a Geriatric Day Hospital, in which case, the day of the following attendance of the Day Hospital was chosen for the patient to be assessed by a member of the LOAP. 

#### 3.2.2. Contacts

The workload between doctors and nurses is rather evenly distributed within the team (Table 2). However, we have observed some variability in the distribution of the workload over the analysed 5-year period, and this reflects both the temporal changes in the team composition (e.g., prolonged sick leave, having a full time clinical trainee) and the flexibility of the team members to accommodate these changes so that the team continues to provide undisturbed quality of clinical care. 

The majority of the referred patients (up to 86% in 2009/2010 year) had the first assessment with the liaison nursing team. However, over the last 2 years of service activity, there was an increase in joint (doctor/nurse) first clinical assessments (18%-19% for the 4th and 5th year). This is largely due to the complexity of the referrals, requiring multiple immediate decisions regarding diagnosis, urgent medical treatment, and behavioural management, as well as providing support and further education about the management of acutely mentally disturbed medically ill individuals. 

Although at the beginning of the LOAP service, there was a substantial clinical activity regarding the need for telephone triage of referrals (25.2%; 22% done by nursing versus 3.2% by medical staff; Table 2), the telephone triage was substantially reduced to 7.4% in 2008-2009 and even further in the last year to 2.3% (Table 2). Similarly, the number of referred patients who were not seen dropped down significantly in 2010 [from 16.5% (37/731) in 2005 to 2.4% (*n* = 19) in 2010]. This may largely be due to improving of the response time (as discussed above), and the service users becoming more familiar with the clinical activities of the LOAP service over time.

#### 3.2.3. Reviews

 In the first year of the establishment of the LOAP service, 16% of all referred patients were not seen (discharged before being seen, mental problems resolved before being seen, advice given over the telephone, or inappropriate referrals needing involvement of other service, etc.). Using the telephone triage, we identified additional 66 patients that did not need assessment (total number of 184, 25.2%). The rest of the patients were reviewed on average 1.8 times. However, when patients who have been seen only once (e.g., for capacity, level of care and/or transfer), were not included in the analysis, the remaining 306 patients were seen on average 2.4 times. Over the following 4 years, this figure remained largely unchanged, with the latest analysis showing that the majority of referred subjects (up to 59%; 442/789) requiring 3 or more followups, after the initial assessment. This suggests that the LOAP provides intensive followup of medically ill patients referred to the service whilst they are inpatients.

### 3.3. Referrals to LOAP

Over the 5 years LOAP activity the reasons for referrals did not change substantially, with some patients requiring two or more assessments (average 1.3 requests per patient; 1.1–1.6/patient) (Table 3). The most frequent reasons for referral were mood, level of care, assessment of cognition, medication advice, and behavioural changes. Over the 5-year analysed period, there were no major changes with respect to the observed reasons for referral for mood and anxiety, level of care, medication advice, and behavioural problems. However, the biggest change in the LOAP activity was the increased number of referrals for cognitive impairment, from 19% in the first year to 49% (Table 3). 

Usually the assessment outcome reflected the reason for referral. In a more detailed analysis for the first year of the LOAP activity, our assessments detected twofold higher social issues and needs than those noted in the referral notes (Table 3). The outcome measures of level of care and capacity assessments were somewhat lower than the reason for referral, but did not reach significant level. Telephone triage alone resolved 16.1% of the LOAP referrals.

## 4. Discussion

This study outlines several important clinical activities undertaken by the LOAP: (i) most referred patients (up to 86–92%) are seen and assessed for their mental health needs within one day of contacting the LOAP team; (ii) majority of first clinical assessments are done by the nursing liaison team; (iii) the LOAP team facilitates in identifying a high rate of social problems in the medically ill elderly. Additional findings include the following.

1. High referral rates for mood disorder (28%), similar to previous studies which reported a higher rate of referrals to both liaison services in general (25.9%; [8]) and liaison psychogeriatric services (18.4%–30%; [9, 10]).
2. Significant increase in referrals for cognitive assessments (from 19% to 49%), supporting the implementation of the National Dementia Strategy [5], enabling a rapid access to specialised mental health services, and facilitating early diagnosis and treatment for the newly diagnosed dementia subjects.
3. Underrepresentation of delirium (1.6–3.3% referral rates), due to delirium being treated actively by physicians, with only complicated cases with prolonged delirium referred to the service. However, we cannot exclude the possibility that some forms of delirium (particularly subsyndromal and hypoactive states) can go undetected.
4. Lowering referral rates for mental capacity assessments (from 11% to 6%). These rates are similar to a previous report that included also younger medically ill (age range 17–91 years of age; [11]). The introduction of the Mental Capacity Act 2005 [12] resulted in lower referral rates, since many of medical/surgical wards conduct mental capacity assessments, and make referrals to the LOAP service only for complex mental capacity issues, or when second opinion is needed.
5. Higher rate of detection of social issues in the elderly medically ill patients. This highlights the need for specific social services input to the LOAP team. In the clinical setting, this is further complicated by a lack of social workers with sufficient knowledge and experience in dealing with medical and mental health problems. This warrants the development of educational programmes to address these issues and integrate them into field practice [13].

In the first year of the establishment of the LOAP service, the telephone triage screening played an important role in identifying 25% referrals that can be dealt this way. However, with reducing response time, and the LOAP service getting known within the medical milieu, unnecessary referrals appear to have been substantially reduced. The educational activities undertaken regularly by all members of the LOAP team, and targeted towards various service users (nurses, social workers, occupational and physiotherapists, trainee doctors, medical and nursing students) may have also contributed to changing the culture dealing with the elderly with mental health problems in medical environment [4]. 

Similarly to a previous study conducted with adults [14], our findings confirm both a greater turnover and intensity of followup over a short period of time by LOAP. Most importantly, the data also provides further evidence that Newcastle LOAP has accepted a liaison (proactive) model rather than a consultation (reactive) model of service delivery, as demonstrated by the intensity of followup per person (up to 3 times/patient), and the increase of assessments in relation to requests (14.1%, as based on the 2005/2006 audit). However, this figure represents an underestimation of our activity, since in a recent audit, undertaken by our team, we have confirmed a high compliance with the NICE guidelines for dementia [15] and depression [4], with 80–100% and 70–100% of referred subjects being routinely screened for cognitive impairment, and depression, respectively (Mukaetova-Ladinska et al., unpublished data). 

In this study, two-thirds of all initial LOAP assessments were done by the liaison nursing team. Liaison nurses' role is not only in the routine clinical assessment, but also being actively involved in triage of medically ill patients, that requires at times telephone prescreening of referrals. We found the latter useful for clarifying the appropriateness of referral to LOAP, the degree of urgency of the referral, as well as obtaining more detailed clinical information. Our liaison nurses, similar to the medical members, also liaise with other services involved in patients' care, and also obtain additional relevant collateral information. Surprisingly, a recent UK survey found that dedicated liaison psychiatry nurses for older people were engaged in only 14% of the services [1]. Based on our analysis, the liaison psychiatric nursing team provides valuable support to LOAP services, enabling prompt delivery of clinical care on 4 hospital sites and 63 inpatient wards. 

Only limited data is available to assess the value of liaison psychiatric nursing input into the medical care of elderly individuals. A randomised control trial on a nurse-led mental health liaison service for older people failed to find a reduction in general psychiatric morbidity, but reported a modest effect on depression [16]. The main contributions of a liaison nursing team seem to be facilitating effective discharge planning and continuity of care [17], and improving staff nurse care for older patients [18], as documented by the intensive followup provided by the liaison LOAP team over a short period of time (majority of patients being seen 3 or more times). Another study demonstrated that the nursing component to the liaison team, besides facilitating access of general hospital patients to specialist mental health care services, is particularly helpful for its focus on practical and care-oriented interventions [19]. This is also the case for our service, where up to 40% of the clinical case load was due to level of care and management of behavioural problems, in which instances the liaison nursing rather than the medical team are involved. Further studies are now needed to evaluate the liaison nursing staff contribution to effective LOAP service in other areas of nursing activity, including counselling, liaison with families, psychological support to patients, their relatives and medical staff, as well as educational support.

In contrast to general adult liaison services [14], LOAP did not have a major portion of urgent referrals. This can only be explained by the LOAP team's rapid response (86–92% of all referred patients seen within one day). Our report, similar to a previous study conducted on liaison adult services [14], confirms greater turnover of patients, as well as more intense clinical involvement over a brief period of time having an average workload of 30–46 patients at any one time for the LOAP service. 

A recent metareview on liaison psychiatric services outlined the need for more evidence-based research to guide liaison service development and planning [20]. The findings of our study need to be taken into account in further Old Age Psychiatry (OAP) service development, which should acknowledge LOAP clinical activity in the context of the full clinical service when making provision for the further development of OAP services and care pathways. Development of good collection data tool will be useful as a guide to service developments. Casemix has been reported to be such tool, which can also provide information about costs associated with the case of medically ill patients with mental health problems [8]. However, this tool has also its limitations (e.g., cannot provide information about change of diagnosis and treatment, recording of follow-ups and outpatient assessments, etc.). Although already accepted in Australia, it remains to be seen whether this or similar tools can be used successfully in monitoring the LOAP activity in the UK. 

## Figures and Tables

**Table 1:** Response time for seeing patients referred to the LOAP. *Data refers to 3-month period only. Please note that for the period 2008-2009 and 2009-2010 only 3-month audits were completed on the LOAP response time.

Response times	2005-2006	2006-2007	2008-2009*	2009-2010*
Same day	287 (39.3%)	191 (31.0%)	67 (55.8%)	118 (56.5%)
Next day	209 (28.6%)	143 (23.2%)	44 (36.7%)	64 (30.6%)
2 days	127 (17.4%	51 ( 8.3%)	11 ( 9.2%)	12 ( 5.7%)
3 days	57 ( 7.8%)	68 (11.0%)	6 ( 5.0%)	5 ( 2.4%)
4 Days	31 ( 4.2%)	41 ( 6.7%)	2 ( 1.7%)	5 ( 2.4%)
5 days	7 ( 0.9%)	26 ( 4.2%)	0	1 ( 0.5%)
Over 5 days	13 ( 1.8%)	73 (11.9%)	0	4 ( 1.9%)
Unknown	—	23 ( 3.7%)	0	0
Total	731	616	120*	209*

**Table 2:** Break down of first contact by members of the LOAP team. F/F: face to face, T/C: telephone contact. *refers to joint (doctor/nurse) assessment (*n* = 188; 18.7%; *n* = 121, 15.3% for 2008-2009 and 2009-2010 year, resp.). **During this period there was a full time clinical trainee attached to the service.

Period	T/C Nurse	F/F Nurse	T/C Doctor	F/F Doctor	No reported contact	Total F/F		
						Nurses	Doctors	Total
2005-2006	161 (22.0%)	321 (43.9%)	23 (3.2%)	226 (30.9%)	0	482 (65.9%)	249 (34.1%)	731 (100.0%)
2006-2007	74 (12.0%)	182 (29.6%)	20 (3.3%)	315 (51.1%)	25 (4.0%)	256 (41.6%)	335** (53.4%)	616 (96.0%)
2008-2009	50 (5.0%)	543* (54.0%)	24 (2.4%)	179* (17.8%)	21 (2.1%)	593* (59.0%)	203* (20,2%)	1005 (97.9%)
2009-2010	0 (0.0%)	546* (69.2%)	18 (2.3%)	85* (10.8%)	19 (2.4%)	546* (69.2%)	103* (13.1%)	789 (97.6%)

**Table 3:** Reasons for referrals and completed assessments. ^‡^Discharged before referral received. Please note that the statistical analysis refers only to the referred and assessed rates for 2005/2006 year. For this, we have used nonparametric analysis (Chi-square test) to test for the differences between referred and assessed sample proportions. Only significant values are presented in the table. *N*  refers to number of referrals.

Requested assessment	2009/2010 referrals (%) *N* = 789	2008/2009 referrals (%) *N* = 1005	2005/2006 referrals (%) *N* = 731	2005/2006 assessments (%)	*χ* ^2^ test *P*-value
Mood	259 (32.8%)	259 (25.8%)	209 (28.6%)	205 (28.0%)	NS
Future care	166 (21.0%)	167 (16.6%)	182 (24.9%)	163 (22.3%)	NS
Cognition	385 (48.8%)	282 (28.1%)	139 (19.0%)	126 (17.2%)	NS
Medication advice*	136 (17.2%)	75 (7.5%)	116 (15.9%)	143 (19.6%)	NS
Behavioural problems	131 (16.6%)	103 (10.3%)	109 (14.9%)	100 (13.7%)	NS
Capacity	63 (8%)	57 (5.7%)	79 (10.8%)	60 (8.2%)	NS
Psychosis	32 (4.1%)	16 (1.6%)	48 (6.6%)	40 (5.5%)	NS
Diagnosis	20 (2.5%)	32 (3.2%)	47 (6.4%)	36 (4.9%)	NS
Delirium	20 (2.5%)	16 (1.6%)	24 (3.3%)	23 (3.2%)	NS
Anxiety	32 (4.1%)	18 (1.8%)	20 (2.7%)	17 (2.3%)	NS
Suicidality	18 (2.3%)	7 (0.7%)	13 (1.8%)	11 (1.5%)	NS
Social issues	0 (0.0%)	0 (0.0%)	60 (8.2%)	124 (17.0%)	*χ* ^2^ = 25.37; *P* = 0.001
Not seen	19 (2.4%)	21 (2.1%)	3 (0.4%)^‡^	118 (16.1%)	*χ* ^2^ = 125.72; *P* = 0.001
Other	20 (2.5%)	31 (3.1%)	11 (1.5%)	19 (2.6%)	NS

Total requests/assessments	1272	1065	1038	1185	*χ* ^2^ = 3.731; *P* = 0.0534
